# Experimental selection of the initial dissolution treatment temperature range for the subsequent cold rolling of IN907 superalloy sheet

**DOI:** 10.1016/j.heliyon.2022.e10138

**Published:** 2022-08-10

**Authors:** Seyed Sajjad Babaie Sangetabi, Seyed Mehdi Abbasi, Rashid Mahdavi

**Affiliations:** Faculty of Materials & Manufacturing Technologies, Malek Ashtar University of Technology, Tehran, Iran

**Keywords:** IN907 superalloy, Initial dissolution treatment, Simultaneous thermal analysis, Laves phase, Plane strain compression, Shear bands

## Abstract

The present study aims to determine the optimal temperature range of the dissolution treatment for the cold rolling of the IN907 superalloy. Samples of the IN907 superalloy hot-rolled sheet, after phase characterization by x-ray diffraction (XRD) and simultaneous thermal analysis (STA) experiments, were subjected to dissolution treatment in the temperature range of 940 °C–1000 °C, followed by cooling in air. The samples were then examined for microstructure, microhardness, tensile properties, and plane strain compression (PSC). Unlike the XRD test, the STA curve of the alloy showed two exothermic peaks, the first peak in the 750–950 °C temperature range associated with the intragranular laves phase and the second peak in the 1100–1175 °C temperature range associated with the oxide phase. The results showed that with increased temperature of the initial dissolution treatment from 980 °C to 1000 °C, the complete dissolution of the grain boundary laves phase led to an increase in the average grain size from 45μm to 57μm and a decrease in the yield and tensile strength by 42 MPa. The microhardness test showed that increasing the temperature of the initial dissolution treatment had little effect on the microhardness. Also, the flow stress diagrams and the normalized work hardening rate of the PSC test showed a similar behavior despite the observation of shear bands in the microstructure center of the compressed sample.

## 1 Introduction

IN907 superalloy (UNS N19907) is a Fe–Ni–Co-based alloy known for its low thermal expansion coefficient, excellent resistance to hydrogen embrittlement, and high strength to a maximum temperature of 650 °C [1]. This superalloy is usually processed by thermomechanical processes and used in aerospace applications, including gas turbine engine components such as shells, seals, and rings [2]. As the IN907 superalloy is sensitive to thermomechanical processing and associated anisotropy, it is essential to observe the plane strain conditions. The PSC test is used to simulate the thermomechanical process with plane strain conditions physically. This experiment can simulate the yield conditions in rolling, and it can prevent lateral expansion and create plane strain conditions by increasing the homogeneous yield stress factors [3].

Two critical data can be extracted from the PSC test: The effective stress-strain curve used to study the flow behavior of the metal and the work hardening rate-effective strain curve used to study the work hardening behavior of the metal. Since interpreting the effective stress-strain curve is difficult due to unintended technological errors, the latter is discussed in more articles [4, 5]. Traditionally, the tensile test is the mechanical test of choice to describe the stress-strain response. However, due to the industrial importance of higher strains, more detailed studies of microstructure evolution are conducted on rolled (or deformed by PSC) materials. A usual defect observed in the rolling process in high strains is a shear band. Shear bands consist of elongated crystals separated by large angular boundaries. El-Danaf et al. [6] reported that these micro-scale bands were the dominant microstructural features in large rolling strains in low SFE FCC metals such as 70/30 brass alloy. The shear bands, which are usually observed in the final regime (regime D) of the normalized work hardening rate curve against the effective strain of superalloys under simple pressure, have a much lower work hardening rate in the same curve and position from the PSC test [6]. However, it is not yet clear whether shear bands occurring in the PSC state have a significant effect on the flow stress response and strain hardening of the IN907 superalloy. A better understanding of the parameters affecting microstructure changes (e.g., a change in the dissolution temperature) on the mechanical responses of this alloy may develop new applications and a unique combination of strength and suitable toughness for the thermomechanical process containing plane strain conditions. This study aims to investigate the change in the temperature of the initial dissolution treatment and its effect on microstructure, microhardness, tensile properties, and PSC to determine the optimal temperature range of dissolution treatment for the cold rolling of IN907 superalloy.

## 2 Experiments

### 2.1 Materials and heat treatment

The material used in this research is the IN907 superalloy. The alloy was melted and cast in a vacuum induction melting (VIM) furnace and then refined via the electro-slag remelting (ESR) process. Table 1 shows the chemical composition analysis by spark emission spectrometer. For this analysis, a 2004 Spectro spark emission spectrometer was used.

**Table 1:** Chemical composition of the used alloy and the standard alloy composition IN907 (Wt %).

Composition	Ni	Co	Nb	Ti	Al	Si	Fe
Standard [7]	35–40	12–16	4.3–5.2	1.3–1.8	<0.2	0.07–0.35	Remained
Alloy used	35.2	14.1	5.1	1.5	0.11	0.25	Remained

The ingots prepared by hot rolling reached a thickness of 2.5 mm. The final pass strain value to achieve this thickness was approximately ε ≈ 0.1. An initial dissolution treatment was performed on a 2.5 mm sheet in four temperatures of 940 °C, 960 °C, 980 °C, and 1000 °C for 1 h to find a suitable temperature range for cold rolling. The thickness of all samples was reduced by a magnetic grinding machine from 2.5 to 1.7 mm to reduce surface oxidation and increase the accuracy of the research (no effect of thickness on the results).

### 2.2 Microstructure characterization

#### 2.2.1 Scanning electron microscope (SEM) and optical microscope (OM)

After preparing for microstructural analysis, the samples were etched in an etching solution mixture of 16 gr FeCl3, 50 ml HCl, and 1 ml HNO3 [8]. An Olympus BX51 optical microscope was used to observe the structure of the samples, and a Vega TESCAN electron microscope was used for further microstructure examination. An EDS analyzer system was used to investigate the chemical analysis of the matrix structure and laves and oxide phases. The grain size and thickness of the grain boundary secondary phase were calculated by ImageJ image analysis software and the ASTM E112 test [9].

#### 2.2.2 XRD

The XRD analysis was used to evaluate the phases in the microstructure further. For this experiment, a copper anode with λ = 1.54 α was used. The ICOD reference cards with 0908–017-00 and 1417-047-00 codes in Plus Xpert Highscore software were used to identify the laves phases and γ matrix, respectively.

#### 2.2.3 STA

As no phase peak with a volume fraction below 5% (such as the oxide phase in the XRD test) was observed, the STA test was used. This experiment was performed in an alumina container under atmospheric air conditions, with an ambient temperature of up to 1200 °C and a heating rate of 10 min/°C. The primary purpose of the experiment was to obtain a DTA curve for determining the range of formation and dissolution of secondary phases.

### 2.3 Tests of mechanical properties of ambient temperature

#### 2.3.1 Microhardness

The Vickers microhardness test was performed using the ASTM E384 by an Ogawa Seiki machine with a load of 500 g [10]. This test was repeated 5 times for each sample.

#### 2.3.2 Tensile

Tensile testing was performed using the ASTM E8 [11] by Instron 8502 with a jaw speed of 1 mm/min and a strain rate of 0.028 s^−1^. A subsize specimen with a gauge length of 12.5 mm, a thickness of 1.7 mm, and a width of 3 mm was used for the experiment. Test specimens were set in the longitudinal rolling direction. This test was repeated 3 times for each sample.

#### 2.3.3 PSC

The PSC test was performed on samples with dimensions of 1.7 × 20.1 × 30 mm using Instron 8502 with a capacity of 10 tons, jaw speeds of 0.50, 0.65, 13 mm/min, and strain rates of 0.014, 0.018, 0.368 s^−1^. Molybdenum disulfide was used as an interfacial lubricant [12]. This experiment was used to express the workability behavior of IN907 superalloy by drawing the Van-Mises effective stress-strain diagram [13] and evaluate the work hardening rate by drawing a normalized work hardening diagram [6]. The Van-Mises relationship was used to convert the experiment data into effective stress-strain [13]. The plane strain conditions ($\varepsilon_{1}=-\varepsilon_{3}،\;\varepsilon_{2}=0$) in the PSC experiment were established by applying geometric constraints ($2\leq \frac{b}{t}\leq 4\;$ and$\;5\leq \frac{w}{b}\leq 12$) [5]. Here, *b* is the width of the compression mold (4 mm), *t* is the thickness (1.7 mm), and *w* is the sample width (20.1 mm). The effective stress and strain during the PSC process can be calculated using Eqs. (1) and (2) [13]:

(1) $$\bar{\sigma }=\frac{\sqrt{3}}{2}P_{a}\;$$

(2) $$\bar{\varepsilon }=\;\frac{2}{\sqrt{3}}\varepsilon_{1}=\frac{2}{\sqrt{3}}\ln\left(\frac{h_{0}}{h_{1}}\right)\;$$

**Figure 1:**
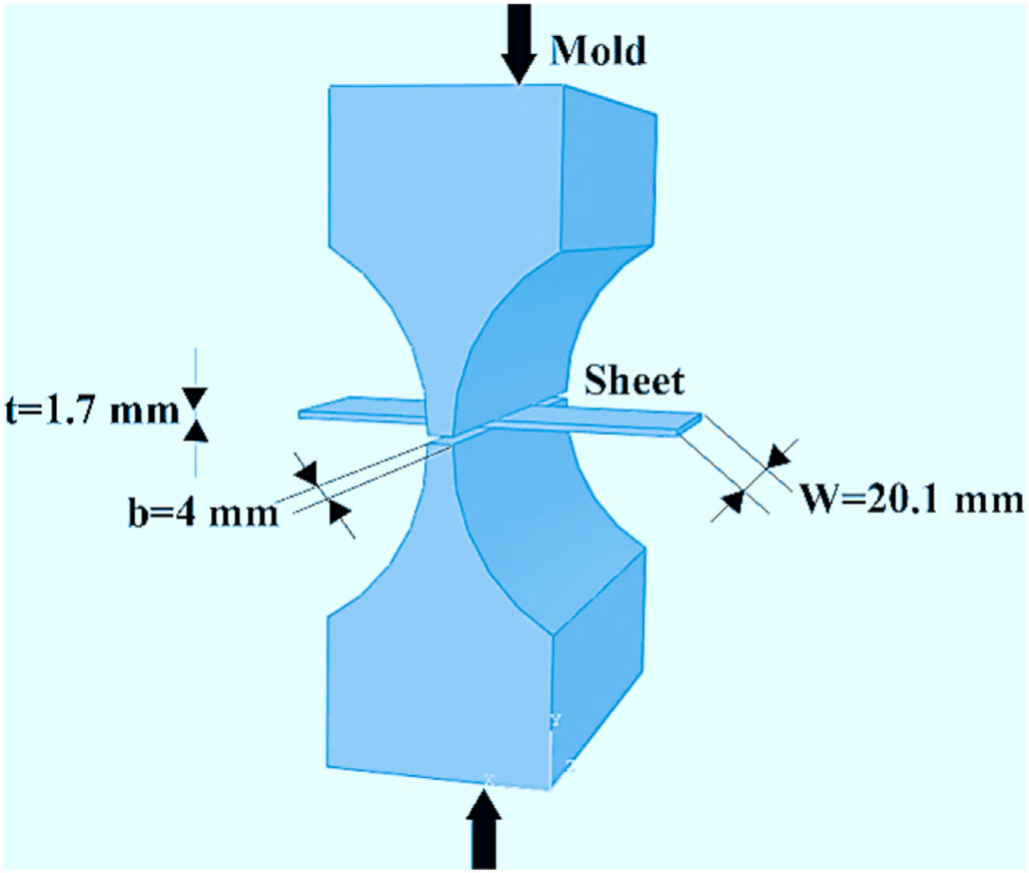
Schematic of the PSC test used.

In the above relation, *P*_*a*_ and *ε*_*1*_ indicate pressure applied to the mold and the strain in the direction of the main axis, respectively. The thickness of the sheet before and after deformation is shown by *h*_*0*_ and *h*_*1*_, respectively. Figure 1 shows the dimensions of the Watsford mold and the sheet used for the PSC test. It is necessary to derive effective stress-strain curves to plot the work hardening rate curve, but short-range noises can complicate such derivation calculations. The noises in the diagram can be removed and presented as four and five-sentence functions using the Origin software and through the smooth option of the Savitzky-Golay method. More details on noise removal can be found in other studies [14].

The work hardening rate is then normalized by the shear modulus (56 GPa). Here, the shear modulus ($G=\frac{E}{2\left(1+\upsilon \right)}$) of the elastic modulus (153 GPa) and the Poisson coefficient (0.36) are obtained from the tensile test specimen and IN907 alloy standard of Special Metals [7]. This test was repeated 3 times for each sample.

## 3 Results and discussion

### 3.1 Identification of phases in the microstructure of the as-received sample

#### 3.1.1 SEM and OM

**Figure 2:**
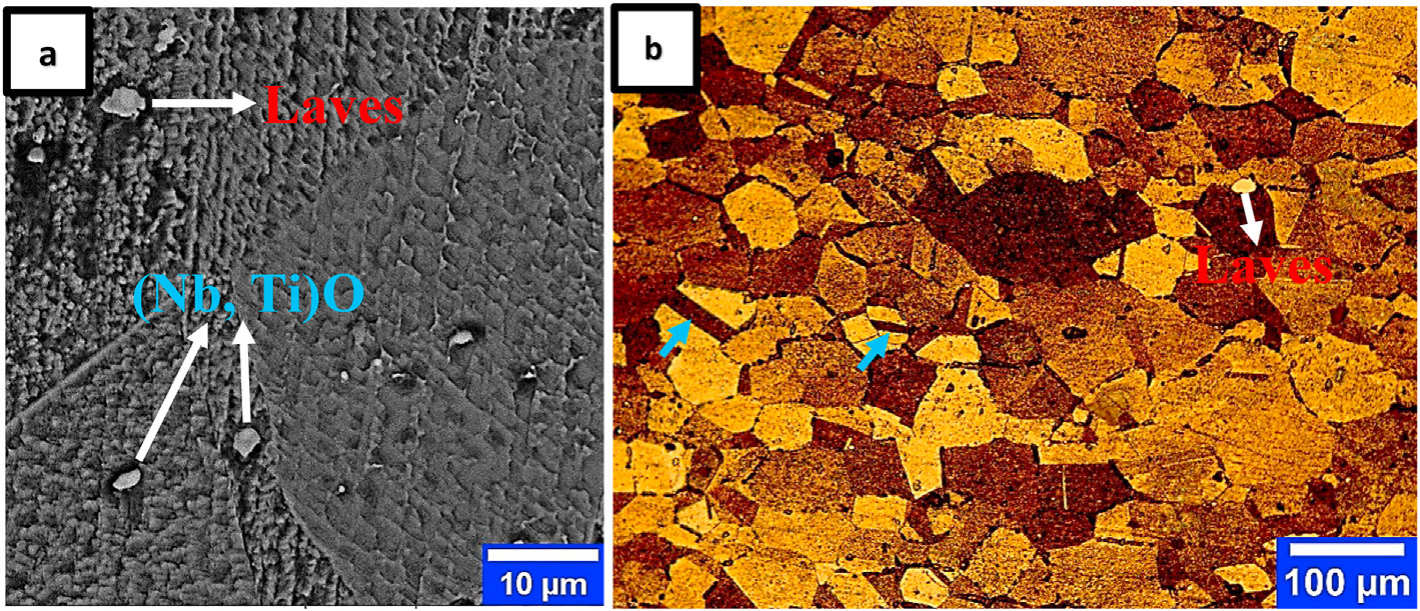
Microstructure of IN907 alloy hot-rolled sheet; a) SEM-BSE, b) OM. The blue and white arrows represent the annealing twins and secondary phase particles in the microstructure, respectively.

Figure 2 shows the SEM-BSE and OM images of the as-received sample after the hot rolling process from two separate areas. As shown in the micrographs, the microstructure consists of a non-uniform fine grain structure with an average grain size of approximately 29 μm. The second phase particles in Figure 2 were also examined using the EDS analysis, and the results can be seen in Table 2. The results showed that in addition to the matrix phase, the laves phases Fe_2_Nb and (Nb, Ti)O are the main phases in this microstructure. Researchers [8] have found that the presence of niobium, silicon, titanium, oxygen, and a high Ti/Si ratio improves the intermetallic phase formation of laves and oxide in iron-nickel-based alloys. The presence of distortion-free annealing twins (Figure 2b, blue arrows) and elongated-free grains in the sheet microstructure (Figure 2b) indicates an FCC-type recrystallized structure without strain. Annealed twins are usually formed during the recrystallization of FCC metals with medium or low SFE, such as austenitic stainless steels and superalloys. However, in FCC dual-phase alloys with medium and high SFE, twins are frequently associated with coarse second-phase particles [15]. In FCC metals with low SFE, the relationship between annealing twin formation and secondary phase particles remains unexplored.

**Table 2:** EDS analysis of the secondary phase particles in the microstructure of IN907 alloy hot-rolled sheet.

ELM(Wt%)	Fe	Ni	Co	Nb	Ti	Si	O
Laves	25.1	27.7	16.7	28.5	0.76	1.1	-
(Nb,Ti)O	1.7	2.1	0.6	82.3	10.4	0.2	3.1

#### 3.1.2 XRD

**Figure 3:**
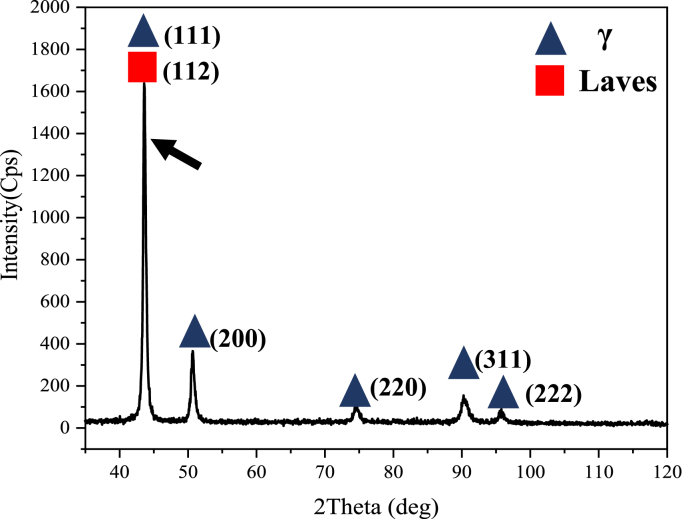
XRD analysis of the as-received hot-rolled sheet of IN907 alloy. The dark arrow indicates the overlap of the laves phase and the matrix.

Figure 3 shows the result of the XRD analysis of the as-received sample. As can be seen, the main peaks of the laves phase and the matrix are overlapping (Figure 3, dark arrow). This overlap, together with the difficulty of detecting oxide phase peaks in superalloys and their dramatic effect on mechanical properties [2, 16, 17], makes the XRD test unsuitable for analyzing this alloy.

#### 3.1.3 STA

**Figure 4:**
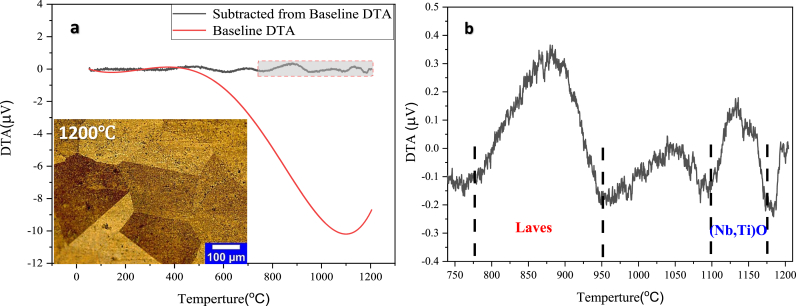
STA diagram of IN907 alloy hot-rolled sheet; a) DTA baseline diagram and reduced baseline diagram with hot-rolled sheet microstructure after STA, b) Magnification of the selected area against temperature changes.

Figure 4 shows the STA (DTA data) result of the hot-rolled sheet heated to 1200 °C for examining the current phases in more detail, especially in the temperature range of the dissolution treatment.

Two separate exothermic peaks were observed during the heating of the IN907 alloy hot-rolled sheet (Figure 4b). Previous research on similar superalloys IN909, IN718, and IN625 [8,18] shows that the first exothermic peak with a temperature range of 775–950 °C is related to the Laves phase, and the second exothermic peak with a temperature range of 1100–1175 °C is related to phase (Nb, Ti)O. These findings are compatible with the microstructure obtained after the completion of thermal analysis at 1200 °C. It can also be seen from Figure 4b that the first peak is higher (in intensity) than the second, indicating [19] a higher volume fraction of the laves phase than the (Nb, Ti)O phase. As expected, grain growth (223 μm) is associated with the approximate dissolution of all secondary phases at 1200 °C. Samples of the hot-rolled sheet were heated at four application temperatures located in the nucleation and dissolution range of the laves phase to compare the STA diagram with the working conditions and select the temperature range of the initial dissolution treatment for the subsequent cold rolling operation. It is considered that the change in temperature of the initial dissolution treatment in the subsequent sub-sections is confirmed by microstructural evaluation and mechanical tests of ambient temperature such as microhardness, tensile, and PSC.

### 3.2 The effect of initial dissolution treatment on the microstructure

**Figure 5:**
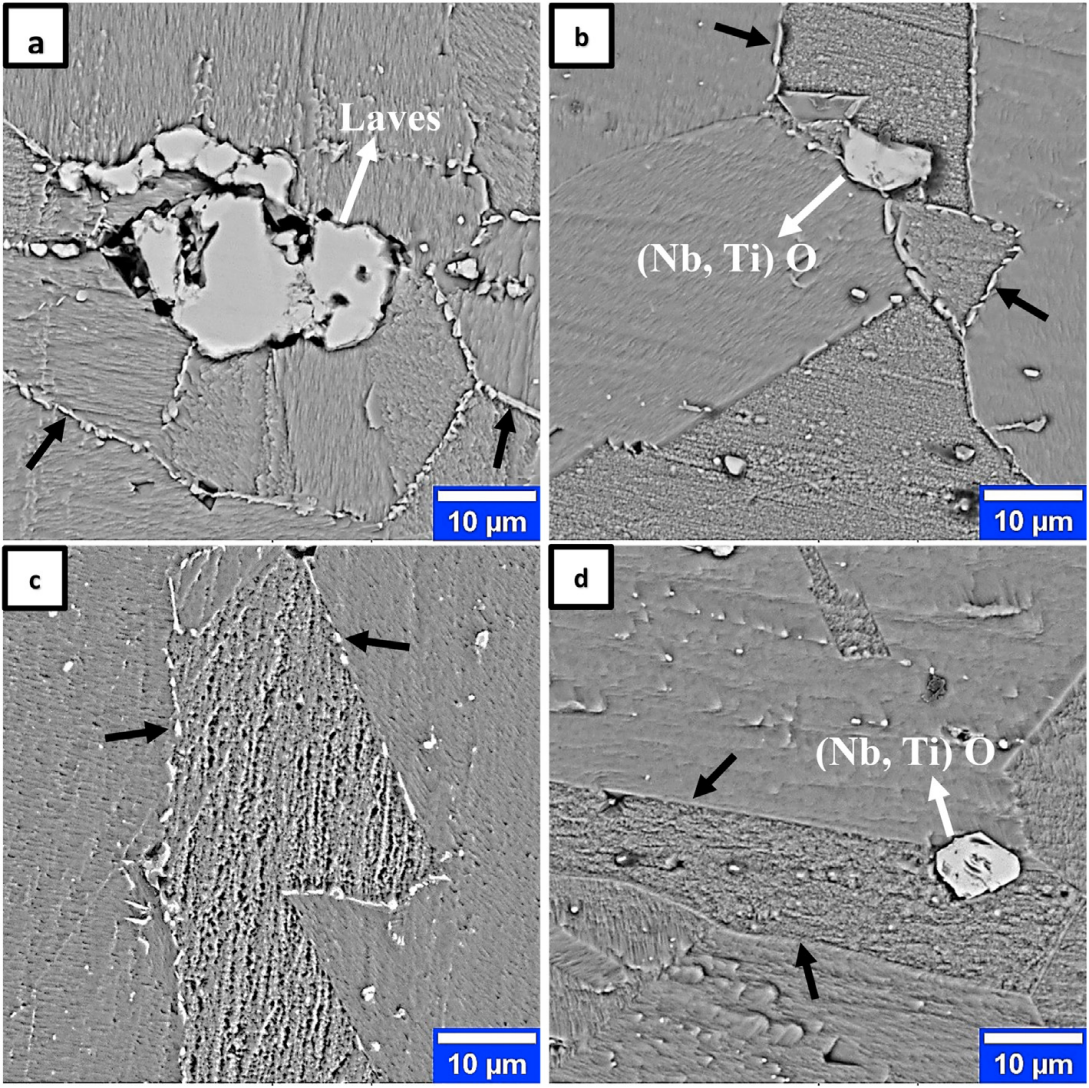
SEM-BSE images of dissolution treated samples of IN907 alloy at temperatures; a) 940 °C, b) 960 °C, c) 980 °C, d) 1000 °C. Dark arrows and white arrows represent the intergranular laves phase and other particles identified from the secondary phases, respectively.

**Figure 6:**
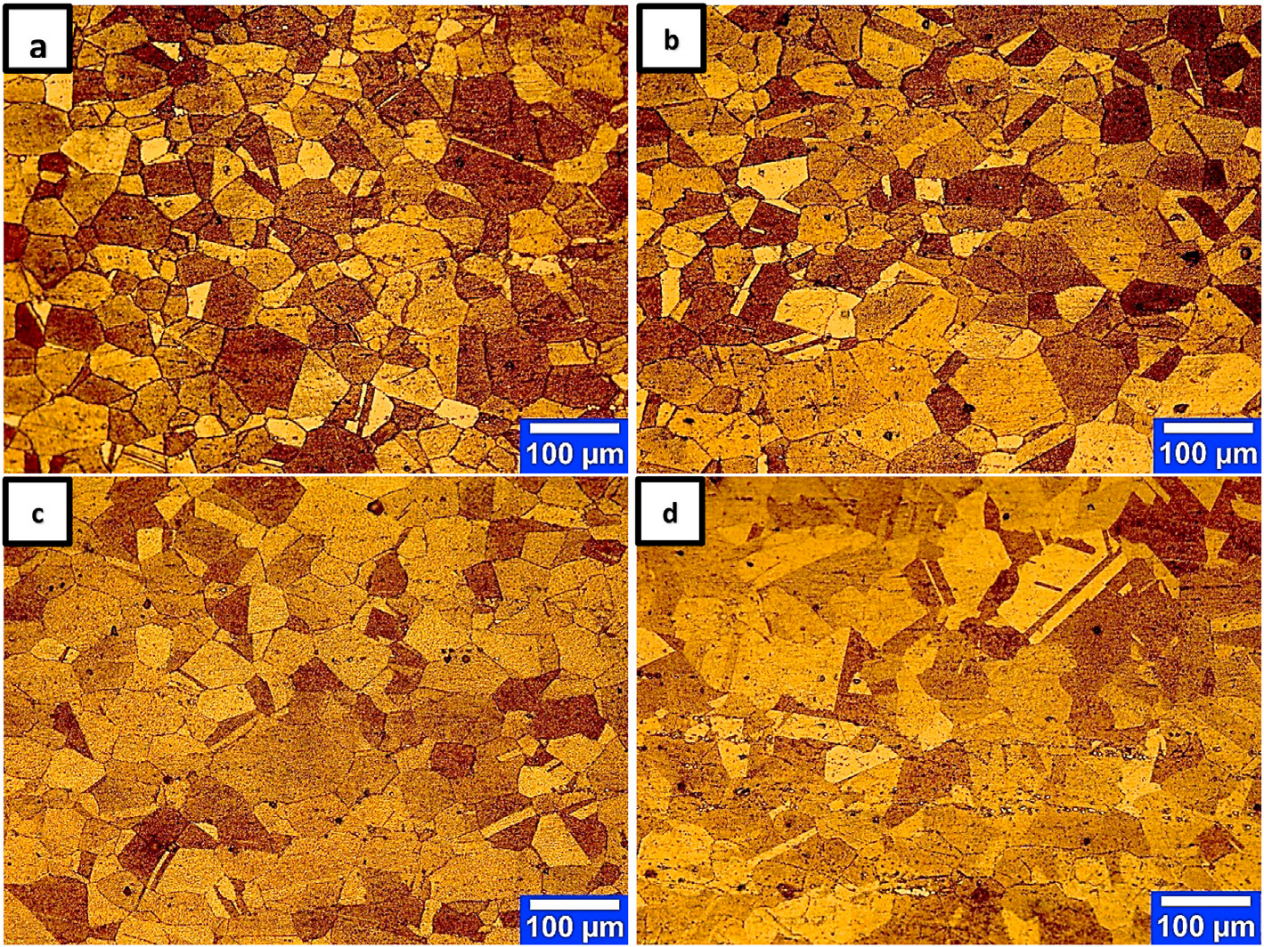
OM images of dissolution treated samples of IN907 alloy at temperatures; a) 940 °C, b) 960 °C, c) 980 °C, d) 1000 °C.

Figures 5 and 6 show the SEM and OM images of samples of dissolution treated at 940 °C, 960 °C, 980 °C, and 1000 °C.

**Figure 7:**
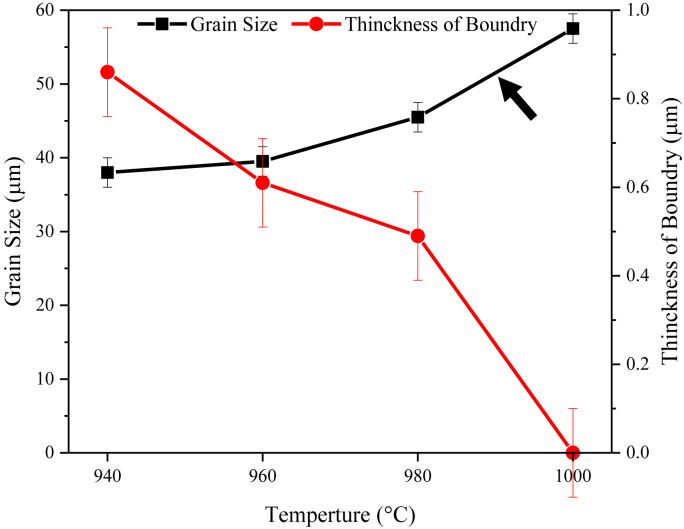
Effect of dissolution treatment temperature on intergranular phase thickness and average grain size of IN907 alloy. The dark arrow indicates an enhancement in the slope of the grain size changes.

The comparison of the dissolution-treated samples at 940 °C and 960 °C (Figure 5 a, b) with the STA diagram (Figure 4b) shows that only the intragranular laves phase is removed at the laves phase dissolution temperature (950 °C in the STA diagram). The intergranular laves phase with partial dissolution remains at 980 °C (Figure 5c) and is eliminated with a further increase to 1000 °C (Figure 5d). The dark arrows in Figure 5 show the intergranular laves phase changes with increasing temperature. This phase is observed at 940 °C, 960 °C, 980 °C, and 1000 °C with continuous, rod, partial dissolution, and complete dissolution morphologies, respectively. The absolute dissolution temperature of the laves phase in the T.T.T diagram of the IN909 superalloy (with approximately 0.2 more Si than the IN907 alloy) is 1040 °C [1]. The cause of the 40 °C reduction compared to the present IN907 alloy [16, 17] is the effect of the Si value on the precipitate temperature range of this phase, which decreases its value to reduce the temperature range of this phase. Brooks et al. [16] showed that increasing Si in A286 alloy causes γ/Laves eutectic formation and decreases its formation temperature. The precise eutectic temperature phases such as γ/Laves and γ/MC for 900 series superalloys have not been determined [18]. However, Knorovsky et al. [16], by examining the DTA results on the IN718 nickel-iron-based alloy, showed that the γ/Laves eutectic temperature is significantly lower than the eutectic temperature γ/MC (1198 °C vs.1298 °C). In particular, Si and C in this alloy increase the probability of forming γ/Laves and γ/MC, respectively. Further examination of Figures 6 and 5 shows that a change in the temperature of the dissolution treatment creates a modifies in the grain size of the austenitic matrix and the thickness of the grain boundary phase. These changes can be seen in Figure 7.

Figure 7 shows that increasing the temperature of the dissolution treatment from 940 °C to 1000 °C led to a decrease in the thickness of the intergranular phase from 0.86 μm to 0 μm. In contrast, the average grain size increased from 38 μm to 57 μm. The decrease in the thickness of the intergranular laves phase and the increase in the average grain size are evident in Figures 5 and 6. As the temperature increases, the driving force for the atoms’ diffusion in the crystal lattice and the growth rate of the grains increase. As a result, the growth rate of austenitic grain size and its rate of change increase with higher temperature and annealing time. The diagram in Figure 7 also changes with a steeper slope, especially at 1000 °C (dark arrow). The basic process during grain boundary migration is the transfer of atoms to or from grains adjacent to the boundary. It is known that the areas of the grain boundaries that are adjacent to the grain boundary concavity are in a compressive state. The areas in the grain boundary arc are in the tensile state. Therefore, the grain boundary motion, which causes the removal of some grains and growth of others, always occurs towards the center of the grain boundary concavity [20]. Grain size and coarseness characteristics are essential in crop production processes because these products need to be optimized for grain size and its control to avoid defects. Several researchers [21, 22] have reported that the dispersion of secondary phase particles inhibits grain growth due to a strong locking force on the migratory grain boundaries at high temperatures. This is confirmed through the absence of secondary phase particles in the heat-analyzed sample up to 1200 °C (Figure 4) with an average grain size of about 232 μm. In the presence of phase particles (Nb, Ti)O, the locking of grain boundaries often occurs by the laves phases present in the structure. This is chiefly due to [19, 22] the higher volume fraction (higher intensity of the laves phase peak in Figure 4) and the more uniform distribution of the laves phase than (Nb, Ti)O.

### 3.3 The effect of initial dissolution treatment on the mechanical properties of ambient temperature

#### 3.3.1 Microhardness

**Figure 8:**
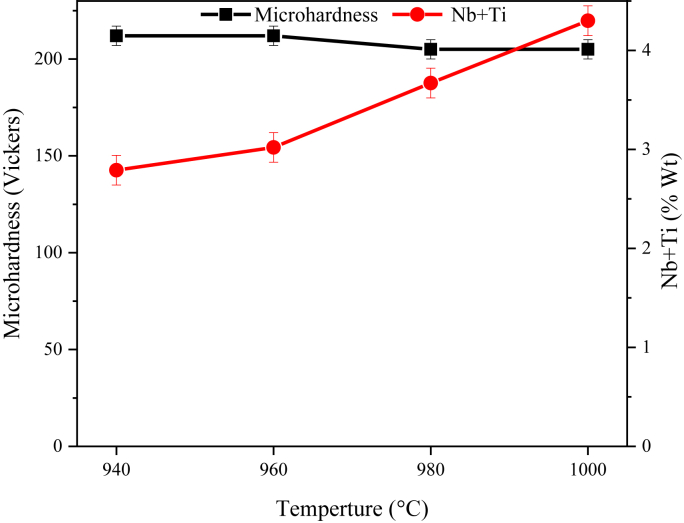
Effect of dissolution treatment temperature on microhardness and weight percentage of Nb + Ti of the IN907 alloy matrix.

Figure 8 shows the changes in microhardness and weight percentage of Nb and Ti-strengthening elements at different temperatures of dissolution treatments. As can be seen, the hardness decreases with increasing the temperature of the dissolution treatment. In contrast, the concentration of solution-strengthening elements with sizeable atomic radius (e.g., niobium and titanium) increases in the matrix solid solution. Unlike IN625 and MP35N superalloys [23, 24], strengthening mechanisms by solid solution and short-range order (SRO) formation do not affect hardness reduction.

The comparison of the results of the present study with the results of the IN907 alloy by X. Y. Y. K. Z. Xu et al. [25] indicates that increasing the temperature of dissolution treatment has a marginal effect on the slope of the hardness diagram of this alloy.

#### 3.3.2 Tensile

**Figure 9:**
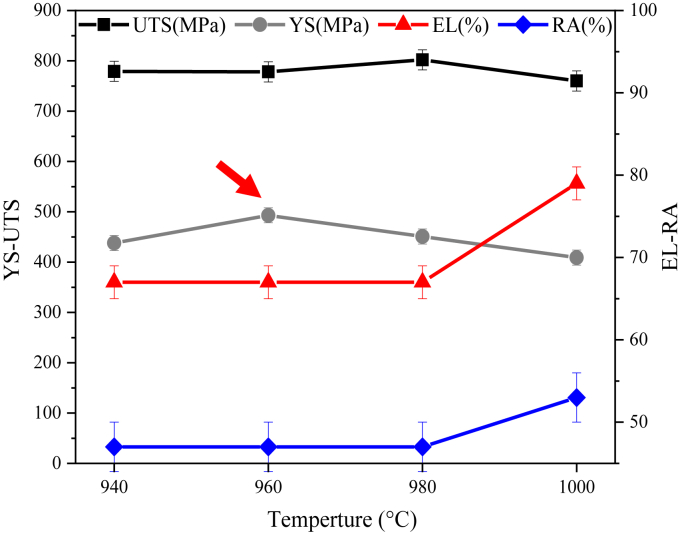
Effect of dissolution treatment temperature on tensile properties of IN907 alloy in ambient temperature. The red arrow indicates the highest level of yield strength.

Figure 9 shows the effect of temperature changes of dissolution treatment on the tensile properties in ambient temperature. As can be seen, increasing the temperature of the dissolution treatment to 980 °C has little effect on elongation, area reduction, and tensile strength.

The lowest strength parameters (409 MPa yield strength and 760 MPa tensile strength) and the highest ductility parameters (53% area reduction and 79% elongation) are observed at 1000 °C (Figure 9). At 1000 °C, grain growth reduces the grain boundary area, and the dissolution of the intergranular laves phase reduces the grain boundary effect. Grain boundaries are effective barriers against the movement of moving dislocations. Therefore, the slip of dislocations occurs more easily as the grains grow [26], resulting in decreased strength and increased ductility at ambient temperature. The tensile properties at 1000 °C are consistent with the Hall-Petch relationship (Equation 3), where the strength decreases with increasing average grain size [27, 28]:

(3) $$\sigma_{y}=\sigma_{0}+\frac{K_{y}}{\sqrt{d}}$$

where *σ*_*y*_ is the yield stress, *σ*_*0*_ is the frictional stress of the material, *k*_*y*_ is the coefficient of strength, and *d* is the mean grain diameter. When the grain size is large, the number of dislocations that can accumulate in a grain behind the boundary increases. In these conditions, the stress concentration increases to activate the slip system in the adjacent grain. Lower external stress is required to yield the adjacent grain, thus reducing the yield stress.

#### 3.3.3 PSC

**Figure 10:**
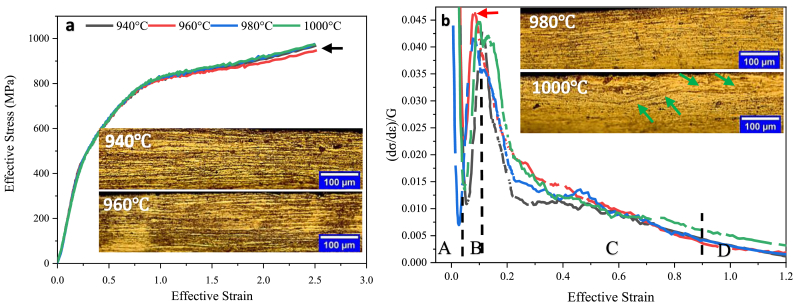
a) Effective stress-strain diagram; b) Normalized work hardening rate-effective strain of IN907 alloy under different dissolution treatment temperatures and constant strain rate 0.014 s^−1^, along with step division and the microstructure of compressed samples after the PSC test. The green, dark, and red arrows indicate shear bands, the lowest flow stress, and the highest work hardening rate, respectively.

Figure 10 shows the effective stress-strain diagram, the normalized work hardening rate-effective strain, and the optical microstructure of the sample center after PSC application under constant strain rate (0.014 s^−1^) and different temperatures of dissolution treatments.

**Figure 11:**
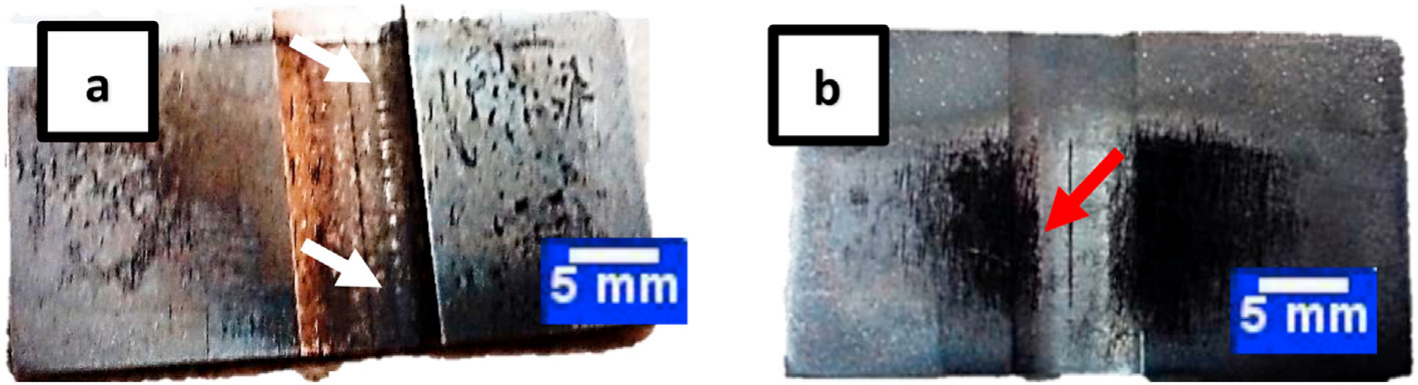
General view of IN907 alloy compressed under ε^O^ = 0.014 s^−1^, macro etched; a) sample representative of 940 °C, 980 °C, and 1000 °C, b) sample representative of 960 °C. The white and red arrows indicate the global crack and the central-local crack, respectively.

As shown in Figure 10a, the flow stress of the specimens continues, reaching large strain amounts with a steady and almost ascending trend. This indicates that increasing the deformation of the material intensifies work hardening, and continuing the deformation requires higher average flow stress. It can be seen in Figure 10a that the dissolution-treated sample shows lower flow stress (approximately 25 MPa) with increasing strain (after strain 1.2) at 960 °C compared to other temperatures (Figure 10a, dark arrow). Unlike the microstructure of different temperatures, a heterogeneous deformation is observed in the microstructure of the dissolution-treated sample at 1000 °C in the center of the compressed sample due to shear bands (Figure 10b, green arrows). As Humphreys et al. [15] have reported for other alloys, this can be attributed to the larger average grain size of this specimen, which has no effect on flow stress diagrams and the work hardening rate. Leffers and Sorenson [6] suggest that the rotation of the plates {111} until an angle is approximately parallel to the rolling plane makes homogeneous slip highly difficult on these plates. This creates an inhomogeneous deformation through shear bands. In the curve of Figure 10b, four separate regimes of normalized work hardening rates for the IN625, MP35N, and IN X-750 superalloys with low SFE values [14, 23, 29] are prominent. As there are no changes in the normalized work hardening rate of regime D, and lines in strains greater than 0.9 strain (end of regime C) overlap, regime D is shown until the strain 1.2. In regime B, unlike other regimes, the strain hardening rate is ascending and relatively constant, which has not occurred with such strength in IN625, MP35N, and IN X-750 superalloys [14, 23, 29] under simple pressure. For further investigation of the curve in Figure 10b, Figure 11 shows an overview of macro etched samples after PSC testing.

Two types of cracks can be seen in the compressed samples under the strain rate of 0.014 s^−1^ (Figure 11). In dissolution-treated samples at temperatures of 940 °C, 980 °C, and 1000 °C, the crack is global (Figure 11a, white arrow), while in dissolution-treated samples at 960 °C, the crack is central-local and about half the length of the other samples (Figure 11b, red arrow). Hua et al. [14] have shown that the dense distribution of deformation twins enables them to occupy a relatively large portion of plastic deformation and form a single grain in different regions. As a result, the stress concentration is relaxed by the operation of the twinning new systems so that no cracks are observed in the deformed specimens under all conditions. The large strains can be attributed to twinning. Thus, the reduction in crack length, the 25MPa decrease in flow stress (Figure 10a dark arrow), and the 0.0025 increase in work hardening rate in regime B (Figure 10b, red arrow) can be attributed to the formation of higher densities of the primary mechanical twins at 960 °C. A similar result is observed in the tensile test with the highest yield stress (Figure 9, red arrow) at 960 °C.

The results show that selecting the temperature range 960–980 °C results in the lowest microstructural defects and the highest mechanical properties for the cold rolling of the IN907 superalloy sheet. This finding allows thermomechanical process designers to process this superalloy sheet with the lowest risk and maximum efficiency.

## 4 Conclusion

The results of microstructure characterization and microhardness, tensile, and PSC tests of the ambient temperature of the sheet IN907 superalloy demonstrate the following conclusions.

1. Matching SEM and OM images with the STA diagram showed that the dissolution temperature of the intragranular laves phase and the oxide phase in the IN907 alloy were 950 °C and 1175 °C, respectively.
2. Increasing the temperature of initial dissolution treatment up to 1000 °C led to an increase in the grain size from 45 μm to 57 μm, which in turn led to a decrease of 42 MPa in yield strength and tensile strength, a 12% increase in total elongation, and a 6% area reduction.
3. Despite the grain growth and fall of tensile properties in the initial dissolution treatment at 1000 °C, the hardness of the alloy at this temperature did not change significantly compared to 980 °C.
4. In the PSC test, increasing the dissolution treatment temperature to 1000 °C led to the formation of shear bands in the microstructure center of the compressed sample. It generally has a marginal effect on the effective stress-strain diagram and the normalized work hardening rate-effective strain.
5. The appropriate temperature range for the initial dissolution treatment for the cold rolling of the IN907 alloy sheet is 960–980 °C.

## Declarations

### Author contribution statement

Seyed Sajjad Babaie Sangetabi: Conceived and designed the experiments; Performed the experiments; Analyzed and interpreted the data; Contributed reagents, materials, analysis tools or data; Wrote the paper.

Seyed Mehdi Abbasi: Conceived and designed the experiments; Contributed reagents, materials, analysis tools or data.

Rashid Mahdavi: Performed the experiments.

### Funding statement

This research did not receive any specific grant from funding agencies in the public, commercial, or not-for-profit sectors.

### Data availability statement

The authors do not have permission to share data.

### Declaration of interests statement

The authors declare no conflict of interest.

### Additional information

No additional information is available for this paper.
